# Serum TFF3 may be a pharamcodynamic marker of responses to chemotherapy in gastrointestinal cancers

**DOI:** 10.1186/1472-6890-14-26

**Published:** 2014-06-14

**Authors:** Li Xiao, Yun-Peng Liu, Chuan-Xing Xiao, Jian-Lin Ren, Bayasi Guleng

**Affiliations:** 1 Department of Gastroenterology, Zhongshan Hospital affiliated to Xiamen University, 201 Hubin South Road, Xiamen 361004, Fujian Province, China; 2 Faculty of Clinical Medicine, Medical College of Xiamen University, Xiangan South Road, Xiangan District, Xiamen 361102, Fujian Province, China

**Keywords:** Gastric cancer, Colorectal cancer, TFF3, Biomarker

## Abstract

**Background:**

As a secreted protein, serum trefoil factor 3 (TFF3) has been reported to be a biomarker of several malignancies. We further investigated whether TFF3 can be applied as a biomarker for and predictor of responses to chemotherapy in gastrointestinal cancer.

**Methods:**

Serum and urine samples were collected from 90 patients with gastric cancer, 128 patients with colorectal cancer and 91 healthy individuals. Serum and urine TFF3 levels were measured using an ELISA.

**Results:**

Serum and urine TFF3 levels were significantly higher in the patients with gastric and colorectal cancer compared with the healthy individuals (*P* < 0.05). Higher serum levels of TFF3 were significantly correlated with distant metastasis and an advanced stage in the two types of cancer (*P* < 0.05). Age and the number of lymph node metastases were significantly correlated with serum TFF3 levels in colorectal cancer, and decreased serum TFF3 levels were significantly correlated with responses to chemotherapy in both the gastric and the colorectal cancer partial response (PR) groups. A combination of serum and urine data did not significantly improve the detection of either cancer, although urine levels have shown a significant negative relationship with the glomerular filtration rate (GFR).

**Conclusions:**

Our data indicate that TFF3 may be an effective biomarker of tumor stage and the presence of distant metastasis, and may be a pharmacodynamic marker of response to chemotherapy in gastrointestinal cancer.

## Background

Gastric cancer is the fourth most common cancer worldwide and has the third highest mortality rate, accounting for 11% of cancer-related deaths [1]. Colorectal cancer is the third most common cancer globally, accounting for 7.6% of cancer-related deaths worldwide [2].

Cancer outcome is highly dependent on the stage at which the disease is detected. Unfortunately, clinical symptoms mostly arise at a late stage, when the disease has already spread outside of the gastric and colorectal region [3]. Surgical excision remains the main treatment, but overall 5-year survival is limited. However, although chemotherapy plays an important role for those patients with advanced gastric and colorectal cancer, conventional chemotherapeutic agents have yielded a treatment bottleneck [4]. Therefore, novel biomarkers for better cancer detection, diagnosis and therapeutic prediction are urgently needed.

Trefoil factor family (TFF) members are small secreted proteins that are co-expressed with mucins by the epithelial cells lining the gastrointestinal tract [5]. In humans, three members of the TFF have been identified, and their functions are thought to center on their role in mucosal protection, namely, their interactions with mucins and the stimulation of cell motility [6-8]. TFF3 or intestinal trefoil factor (ITF) is expressed in the goblet cells of the intestine and shows limited expression outside of the gastrointestinal tract, in the breast, salivary gland, hypothalamus and respiratory tract [6].

TFF3 is overexpressed in a variety of human malignancies, including gastric and colorectal cancer, and has demonstrated prosurvival, proinvasive and proangiogenic activities [9,10]. Secreted proteins can be used as biomarkers to identify and characterize diagnosis, prognosis and potential therapeutic responses. Body fluids such as plasma, serum and urine, tissue specimens and cancer cell lines have been utilized extensively toward this goal [11].

As a secreted protein, serum TFF3 has been reported to be a biomarker for several malignancies [12-16]. In this study, we investigated whether serum and urine TFF3 levels could be applied as biomarkers for gastrointestinal cancer and predictors of responses to chemotherapy. Our data demonstrated that serum TFF3 can be applied as an effective biomarker for the detection of tumor stages and distant metastasis and pharamcodynamic marker of responses to chemotherapy in gastrointestinal cancer. Urine TFF3 is a different indicator than serum levels and could also be a biomarker for the early detection of renal dysfunction.

## Methods

### Ethics statement

This study was approved by the Ethics Committee (No: 20081009) of Zhongshan Hospital, affiliated with Xiamen University in Xiamen, Fujian Province, China. Written consent was obtained from all participants who were involved in this study.

### Patients and healthy control characteristics

In total, 90 patients with gastric cancer, 128 patients with colorectal cancer and 91 relatively age- and gender-matched healthy individuals who underwent health examinations from September 2011 to April 2013 at Zhongshan Hospital were enrolled in this study. Both biopsies and surgical specimens were histologically confirmed to be gastric and colorectal adenocarcinomas. The patients were classified by age, gender, site, the degree of differentiation, lymph node metastasis, distant metastasis and clinical stages. Clinical and pathological information was obtained from a patient database at Zhongshan Hospital, affiliated with Xiamen University. We obtained blood samples from the 218 patients before treatment. Among these patients, 46 with gastric cancer and 57 with colorectal cancer contributed urine samples at the same time. Tables 1 and 2 show the backgrounds of the patients and the clinical characteristics of the gastric and colorectal cancer groups, respectively. In total, 91 healthy individuals (male/female of 51/40 and age of 50.1 ± 9.9 y) were used as controls. The patients in the cancer group were older, and there were a higher number of male patients in the gastric cancer group. Notably, for most populations, these sex differences in gastric cancer are in both high- and low-incidence regions [17]. In this study, 24 patients with advanced gastric cancer and 29 patients with advanced colorectal cancer received chemotherapy. From the chemotherapy group, we obtained blood and urine samples before and after chemotherapy (two cycles later). We then applied the RECIST 1.1 criteria for solid tumors to evaluate the efficacy of chemotherapy.

**Table 1:** Background of gastric cancer patients with serum TFF3 analysis

**Parameters**	**Cases (n)**	**Serum TFF3 (ng/ml)**	***P***
Age			
≤60	44	12.72 ± 1.21	0.0529
>60	46	20.29 ± 3.59	
Gender			
Male	75	16.65 ± 2.28	0.9489
Female	15	16.31 ± 2.90	
Site			
Fundus/Cardia	35	13.96 ± 1.18	
Gastric body	22	20.28 ± 7.43	0.323
Antrum	30	16.92 ± 1.69	
Degree of differentiation			
Well-moderately	41	13.10 ± 1.27	0.1034
Poorly	49	19.51 ± 3.40	
Stage		(I-III *VS* IV)	0.0017
I	9	8.89 ± 1.35	
II	9	12.94 ± 2.24	
III	29	12.07 ± 0.73	
IV	43	22.02 ± 3.89	
Lymphatic metastasis			
N(-)	13	10.71 ± 1.01	0.3057
N(+)	33	12.23 ± 0.83	
N1	12	12.91 ± 1.81	
N2	7	11.84 ± 1.98	0.9603
N3	4	11.85 ± 0.82	
Distant metastasis			
M0	48	11.97 ± 0.76	0.0108
M1	42	21.88 ± 3.97	

**Table 2:** Background of colorectal cancer patients with serum TFF3 analysis

**Item**	**Cases (n)**	**Serum TFF3 (ng/ml)**	***P*-value**
Age			
≤60	70	13.09 ± 0.95	0.0061
>60	58	19.20 ± 2.11	
Gender			
Male	75	16.14 ± 1.42	0.7699
Female	53	15.47 ± 1.82	
Site			
Colon	67	15.86 ± 1.57	0.9957
Rectum	61	15.47 ± 1.82	
Degree of differentiation			
Well-moderately	107	20.66 ± 3.74	0.057
Poorly	21	14.92 ± 1.11	
Stage		(I-III *VS* IV)	0.0007
I	11	11.32 ± 2.21	
II	30	12.20 ± 0.88	
III	25	14.27 ± 2.89	
IV	45	21.82 ± 2.39	
Lymphatic metastasis			
N(-)	46	11.86 ± 0.79	0.1786
N(+)	33	14.70 ± 2.22	
N1	19	10.45 ± 1.09	0.00229
N2	14	20.47 ± 1.98	
Distant metastasis			
M0	84	12.86 ± 0.99	0.001
M1	44	21.59 ± 2.44	

### RECIST 1.1 criteria for the assessment of the efficacy of chemotherapy

Target lesions were evaluated as follows. Complete response (CR): The disappearance of all target lesions. Any pathological lymph nodes (whether target or non-target) must have a reduction in the short axis of <10 mm. Partial response (PR): At least a 30% decrease in the sum of the diameters of the target lesions, using the baseline sum of the diameters as a reference. Progressive disease (PD): At least a 20% increase in the sum of the diameters of the target lesions, using the smallest sum in the study as a reference (including the baseline sum if that is the smallest in the study). In addition to the relative increase of 20%, the sum must also demonstrate an absolute increase of at least 5 mm. Note that the appearance of one or more new lesions is also considered to be progression. Stable disease (SD): Neither sufficient shrinkage to qualify for PR nor a sufficient increase to qualify for PD, using the smallest sum of the diameters in the study as a reference [18].

### ELISA analysis

Serum TFF3 levels were measured by a human TFF3 ELISA kit (R&D Systems, Minneapolis, MN). It is measured using method of double antibody sandwich and antibodies concentration as follow: 360 ug/ml of mouse anti-human TFF3 as a capture antibody and 18 ug/ml of biotinylated sheep anti-human TFF3 as a detection antibody. Capture antibody has been pre-coated onto a microplate. Prior to the ELISA, 1 ml of blood was collected from each healthy individual and each patient, followed by centrifugation for serum separation. All samples and standards were assayed in duplicate. Briefly, the capture antibody was diluted to the working concentration in PBS. A 96-well microplate was coated with 100 μl per well of the diluted capture antibody (2.0 μg/ml). The plate was sealed and incubated overnight at room temperature. The plates were blocked by adding 300 μl of reagent diluents (1%BSA in PBS, PH7.2-7.4) and incubating at room temperature for 1 hour. Next, 100 μl of sample (1 g/l) or standards in reagent diluents were added to each well and incubated for 2 hours at room temperature. The undiluted standard serves as the high standard (100 ng/ml), reagent diluents serves as the zero standard, produce a 2-fold dilution series (1.56 ng/ml, 3.12 ng/ml, 6.25 ng/ml, 12.5 ng/ml, 25 ng/ml, 50 ng/ml) between them. After repeated aspiration and washing, 100 μl of the detection antibody (100 ng/ml) was added and incubated for 2 hours at room temperature. After repeated aspiration and washing, 100 μl of the working dilution of streptavidin-HRP was added to each well and incubated for 20 minutes at room temperature. Next, 100 μl of substrate solution (1:1 mixure of H_2_O_2_ and Tetramethylbenzidine) was added for a 20-minute incubation, followed by the addition of 50 μl of stop solution (2 mmol/l, H_2_SO4). The absorbance at 450 nm was measured. Concentrations of human TFF3 in the serum samples were calculated from the standard curves of recombinant human TFF3.

Urine TFF3 levels were measured by a human TFF3 ELISA kit (Cusabio, IL). This assay employs the quantitative sandwich enzyme immunoassay. Antibody specific for TFF3 has been pre-coated onto a microplate. For this assay, 1 ml of urine was collected from healthy individuals and from patients. Add 100 μl of standards (0 ng/ml, 1.56 ng/ml, 3.12 ng/ml, 6.25 ng/ml, 12.5 ng/ml, 25 ng/ml, 50 ng/ml, 100 ng/ml) and 100 μl of sample (0.08 g/l) per well. After incubation of 2 hours at 37°C, remove the liquid of each well and add 100 μl of biotin antibody (1×) to each well, incubate 1 hour at 37°C, then aspirate and wash 3 times with washing buffer. Add 100 μl of HRP- avidin (1×) to each well and incubate 1 hour at 37°C, then wash 5 times and add 90 μl of TMB Substrate to each well. Incubate 15-30 minutes at 37°C and avoid from light. Then add 50 μl of stop solution and the absorbance at 450 nm was measured. Concentrations of human TFF3 in the urine samples were calculated from the standard curves of recombinant human TFF3.

### Assessment of the glomerular filtration rate (GFR)

In this study, 99 Tc-DTPA clearance was defined as the serum activity of 99 Tc-DTPA at 1 and 3 hours following the injection of 99 Tc-DTPA, and the results were calculated using a constant body surface area of 1.73 m^2^ (ml/min/1.73 m^2^).

### Statistical analysis

Statistical analyses were performed using SPSS v13.0 (SPSS, Chicago, IL), and graphs were generated using GraphPad Prism 5.0 (GraphPad Software Inc., CA). Student’s *t* test and the Kruskal-Wallis test were used to compare data between groups. All values are expressed as the mean and standard deviation (SD), and *P* < 0.05 was considered to be statistically significant. Receiver operating characteristic (ROC) curves were generated to assess the diagnostic accuracy of each parameter, and the sensitivity and specificity of the optimum cutoff point were defined as those values that maximized the area under the ROC curve (AUC).

## Results

### Serum and urine levels of TFF3 were elevated in gastric and colorectal cancer patients compared with healthy individuals

TFF3 is a secreted protein present in the serum and urine and can be detected by ELISA. Therefore, we measured the TFF3 protein levels in serum and urine samples from gastric and colorectal cancer patients prior to treatment, including surgery, chemotherapy and radiotherapy, and from healthy individuals.

**Figure 1:**
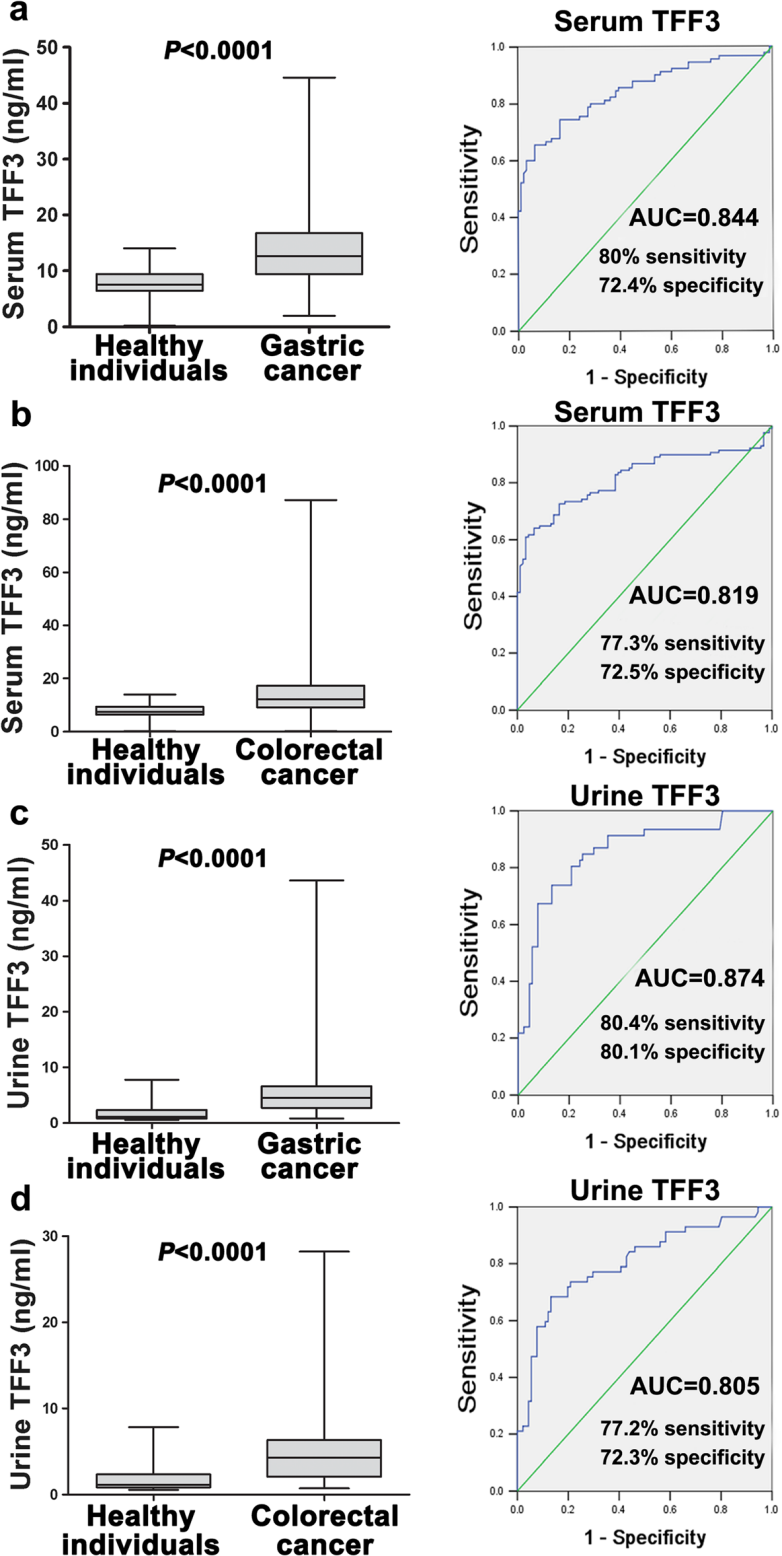
**Measurement of serum and urine TFF3 levels and ROC curve analysis.** With a serum TFF3 levels cut-off values ≥ 8.98 ng/ml, the sensitivity and specificity of TFF3 to distinguish gastric patients from healthy individuals were 80% and 72.4%, respectively. (*P* <0.001) **(a)** With a serum TFF3 levels cut-off values ≥ 8.83 ng/ml, the sensitivity and specificity of TFF3 to distinguish colorectal patients from healthy individuals were 77.3% and 72.5%, respectively. (*P* <0.001) **(b)** With a urine TFF3 levels cut-off values ≥ 3.23 ng/ml, the sensitivity and specificity of TFF3 to distinguish gastric patients from healthy individuals were 80.4% and 80.1%, respectively. (*P* <0.001) **(c)** With a serum TFF3 levels cut-off values ≥ 2.78 ng/ml, the sensitivity and specificity of TFF3 to distinguish colorectal patients from healthy individuals were 77.2% and 72.3%, respectively (*P* <0.001) **(d)**.

The serum TFF3 levels in the patients with gastric cancer were 16.59 ± 1.958 ng/ml, and were significantly elevated compared with 7.80 ± 0.233 ng/ml in the group of healthy individuals (Figure 1a, *P* < 0.05). ROC curve analysis showed that serum TFF3 had 80.0% of positive predictive value, 80% sensitivity and 72.4% specificity, with an AUC of 0.844 and optimal cut-off (8.98 ng/ml) when gastric cancer patients were separated from healthy individuals. The urine TFF3 levels in the patients with gastric cancer were significantly elevated to 6.46 ± 1.046 ng/ml, compared with 1.83 ± 0.162 ng/ml in the group of healthy individuals (Figure 1c, *P* < 0.05). ROC curve analysis showed that urine TFF3 had 73.91% of positive predictive value, 80.4% sensitivity and 80.1% specificity, with an AUC of 0.874 and optimal cut-off (3.12 ng/ml) when gastric cancer patients were separated from healthy individuals.

The serum TFF3 levels in the patients with colorectal cancer were 15.86 ± 1.118 ng/ml, and were significantly elevated compared with 7.80 ± 0.233 ng/ml in the group of healthy individuals (Figure 1b, *P* < 0.05). ROC curve analysis showed that serum TFF3 had 76.6% of positive predictive value, 77.3% sensitivity and 72.5% specificity, with an AUC of 0.819 and optimal cut-off (8.83 ng/ml), when colorectal cancer patients were separated from healthy individuals. The urine TFF3 levels in the patients with colorectal cancer were significantly elevated to 5.56 ± 0.696 ng/ml, compared with 1.83 ± 0.162 ng/ml in the group of healthy individuals (Figure 1d, *P* < 0.05). ROC curve analysis showed that urine TFF3 had 75.4% of positive predictive value, 77.2% sensitivity and 72.3% specificity, with an AUC of 0.805 and optimal cut-off (3.01 ng/ml) when colorectal cancer patients were separated from healthy individuals. However, the combination of serum and urine data could not significantly improve the detection of both cancers compared with serum or urine TFF3 alone (AUC < 0.5), and urine TFF3 did not show a significant correlation with serum levels (data not shown).

Taken together, these data indicated that serum and urine TFF3 levels can be applied as a biomarker for separate gastric and colon cancer detection.

### Serum TFF3 levels is correlated with the development and progression of gastric cancer

**Figure 2:**
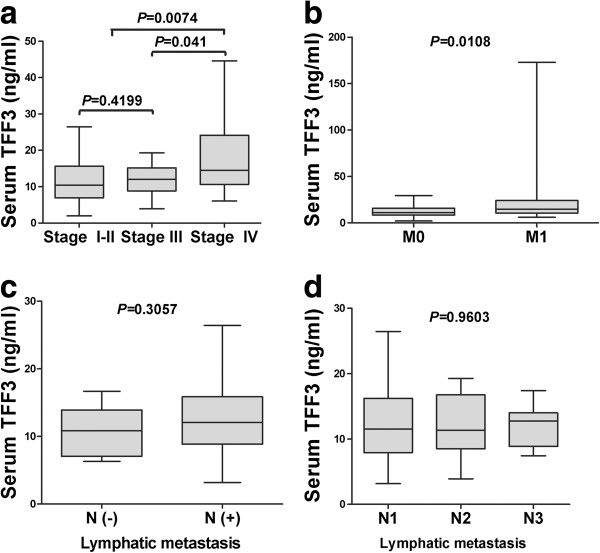
**Measurement of serum TFF3 levels in gastric cancer patients.** Serum TFF3 levels were compared in gastric cancer patients. The significant ratios with P < 0.05 between 2 groups(clinical stage III/IV) and another 2 groups (clinical stage I-II/IV) **(a)** The significant ratios with P < 0.05 between 2 groups M0 (patients without distant metastasis) and M1 (patients with distant metastasis) **(b)**, lymphatic metastasis after excluding the interference of distant metastasis **(c)** and number of lymphatic metastases **(d)**.

To define the prognostic value of serum TFF3 levels in gastric cancer, we analyzed the clinicopathological parameters of gastric cancer. Our data indicated that a higher serum TFF3 level was significantly correlated with the clinical stage (I-III *vs* IV) and distant metastasis (Figure 2a-b, *P* < 0.05). No significant difference was observed in lymphatic metastasis or the number of lymphatic metastases after excluding the interference of distant metastasis (Figure 2c-d). The serum TFF3 level was did not significantly correlated with patient gender or age, the site of the tumor (fundus/cardia, body or antrum) or the degree of differentiation (well, moderately or poorly differentiated) (Table 1).

### Serum TFF3 levels is correlated with the development and progression of colorectal cancer

**Figure 3:**
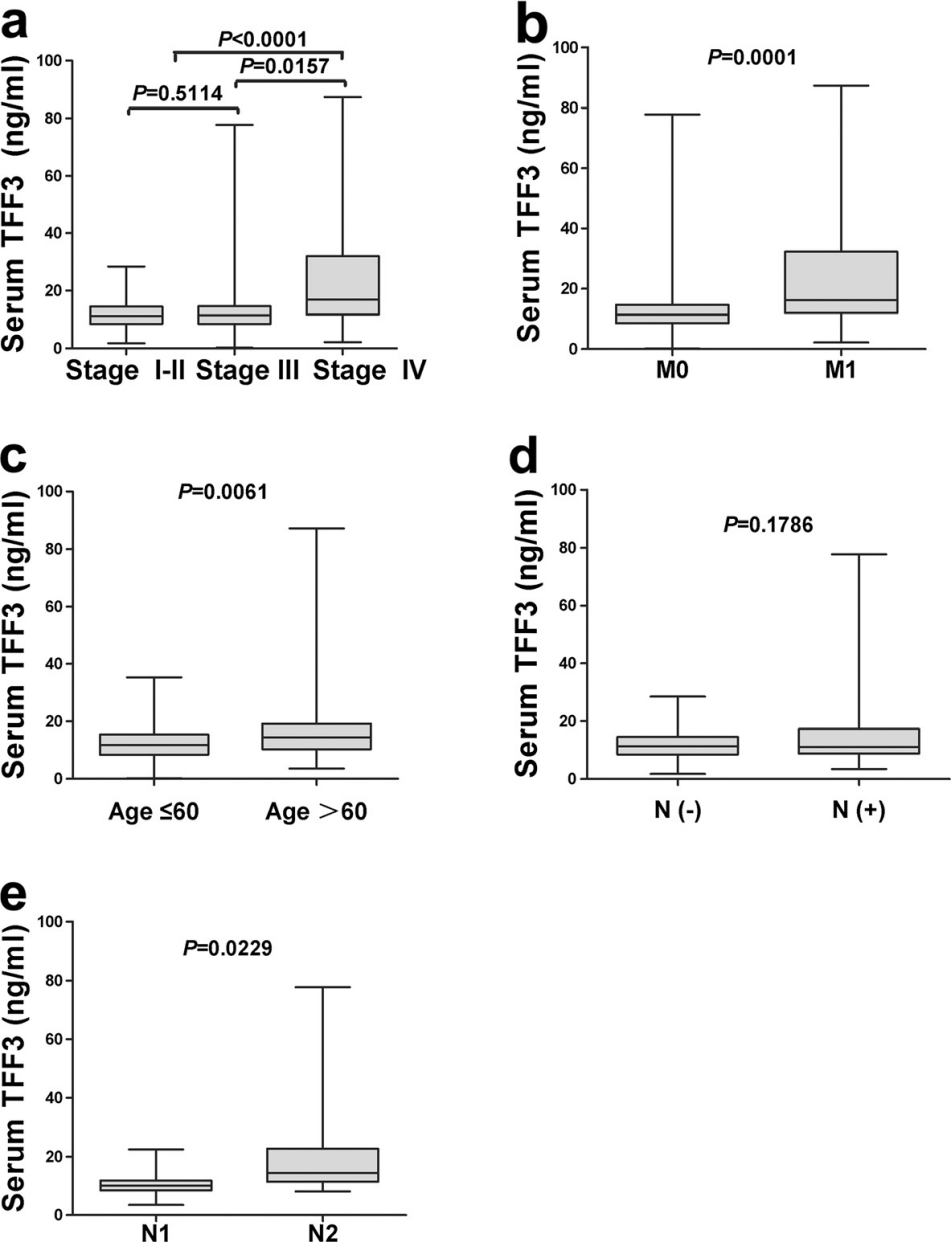
**Measurement of serum TFF3 levels in colorectal cancer patients.** Serum TFF3 levels were compared in colorectal cancer patients. The significant ratios with P < 0.05 between 2 groups (clinical stage III/IV) and another 2 groups (clinical stage I-II/IV) **(a)** The significant ratios with P < 0.05 between 2 groups M0 (patients without distant metastasis) and M1 (patients with distant metastasis) **(b)**, lymphatic metastasis after excluding the interference of distant metastasis **(d)**,The significant ratios with P < 0.05 between 2 groups (age >60 y and age ≤60 y) **(c)** and another 2 groups (N1 and N2) number of lymphatic metastases **(e)**.

To determine the prognostic value of serum TFF3 levels in colorectal cancer, we analyzed the clinicopathological parameters of gastric cancer. Our data demonstrated that a higher serum TFF3 level was significantly correlated with the clinical stage (I-III *vs* IV) and distant metastasis (Figure 3a-b, *P* < 0.05). As shown in Figure 3c, in contrast to gastric cancer, the serum TFF3 levels of older patients (age >60 y) with colorectal cancer were higher than the levels of younger patients (age ≤60 y, *P* < 0.05). No significant difference was observed in lymphatic metastasis after excluding the interference of distant metastasis (Figure 3d). However, different numbers of lymphatic metastases (N1 and N2) showed statistically significant differences (Figure 3e, *P* < 0.05). The serum TFF3 level was not significantly correlated with a patient’s tumor site or degree of differentiation (Table 2).

### Serum TFF3 levels as a pharamcodynamic marker of responses to chemotherapy in both gastric and colorectal cancer PR patients

**Figure 4:**
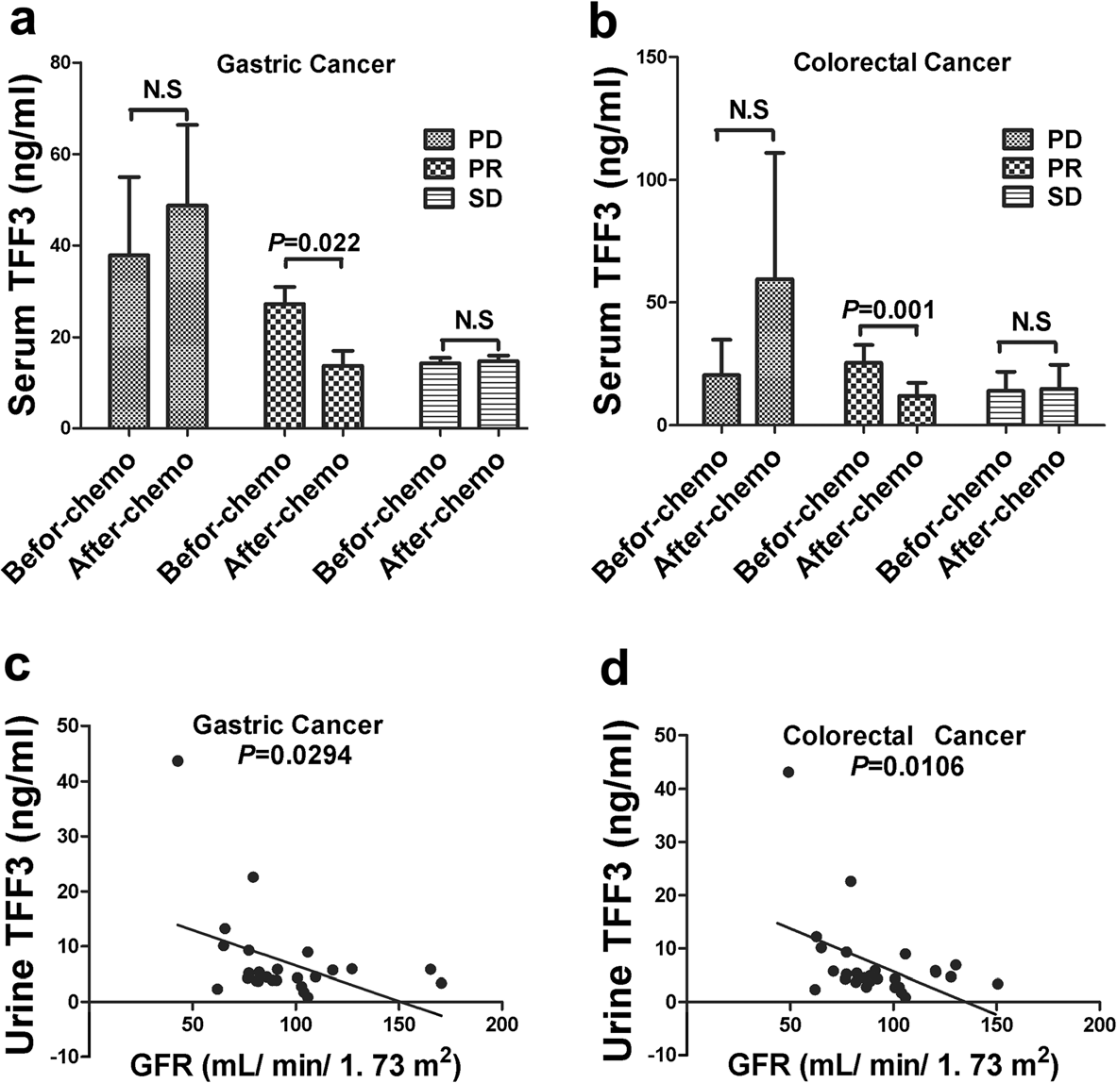
**Serum TFF3 analysis in the chemotherapy group and correlation analysis between urine TFF3 and the GFR. (a)** and **(b)** present an analysis of serum TFF3 levels in the PR, SD and PD groups of patients with gastric and colorectal cancer before and after chemotherapy, respectively. The significant ratios with P < 0.05 between 2 groups (before and after chemotherapy in the PR group of gastric and colorectal cancer). **(c)** and **(d)** present a correlation analysis between urine TFF3 levels and the GFR in patients with gastric and colorectal cancer, respectively. Urine TFF3 levels were significantly negatively correlated with the GFR in both the gastric and the colorectal cancer groups (*P* < 0.05).

To define whether serum and urine TFF3 levels could be applied as predictors of responses to chemotherapy, we analyzed the relationship between serum and urine TFF3 levels before and two cycles after chemotherapy in patients with advanced gastric and colorectal cancer. The chemotherapeutic strategy consisted of fluoropyrimidines (5-fluorouracil, [5-FU]-leucovorin and capecitabine) with oxaliplatin. In total, 24 patients with advanced gastric cancer and 29 with colorectal cancer underwent chemotherapy and were assessed using the RECIST 1.1 criteria. The advanced gastric cancer group showed an efficacy of 0/24 for CR, 6/24 for PR, 9/24 for SD and 9/24 for PD. Significantly higher serum TFF3 levels were observed in the PD group compared with the PR and SD groups. Interestingly, decreased serum TFF3 levels were significantly correlated with responses to chemotherapy in the gastric cancer PR group (Figure 4a, *P* < 0.05). The advanced colorectal cancer group showed an efficacy of 0/29 for CR, 8/24 for PR, 17/29 for SD and 4/29 for PD. Decreased serum TFF3 levels were significantly correlated with responses to chemotherapy in the colorectal cancer PR group. However, no significant differences in serum TFF3 levels were observed between the PR, SD and PD groups (Figure 4b, *P* < 0.05). Taken together, the data indicated that the serum level of TFF3 can be applied as a pharamcodynamic marker of responses to chemotherapy in both gastric and colorectal cancer PR patients.

### Urine TFF 3 levels showed a significant negative relationship with the GFR

We analyzed the relationship of urine TFF3 levels with clinicopathological parameters and responses to chemotherapy and did not find a significant difference between urine TFF3 levels based on clinical stage, lymphatic metastasis, the patient’s gender or age, the site and degree of differentiation of the tumor or responses to chemotherapy for gastric or colorectal cancer (Tables 3 and 4). Therefore, we investigated whether urine TFF3 levels can reflect renal function. As shown in Figure 4c-d, the GFR of 26 gastric and 29 colorectal cancer patients was assessed, and urine TFF3 levels were significantly negatively correlated with the GFR in both the gastric and the colorectal cancer groups (*P* < 0.05).

**Table 3:** Clinicopathological parameters and responses to chemotherapy with urine TFF3 in gastric cancer patients

**Parameters**	**Cases (n)**	**Urine TFF3 (ng/ml)**	***P***
Age			
≤60	24	4.76 ± 0.72	0.089
>60	22	8.33 ± 1.99	
Gender			
Male	36	6.82 ± 1.30	0.529
Female	10	5.20 ± 1.21	
Site			
Fundus/Cardia	18	6.26 ± 1.23	
Gastric body	16	8.47 ± 2.54	0.087
Antrum	12	4.10 ± 0.99	
Degree of differentiation			
Well-moderately	**27**	6.35 ± 0.93	0.898
Poorly	19	6.63 ± 2.20	
Stage		(I-III *VS* IV)	0.414
I-II	9	4.15 ± 0.59	
III	5	6.40 ± 4.10	
IV	32	7.12 ± 1.36	
Lymphatic metastasis			
N(-)	6	4.23 ± 0.79	0.743
N(+)	9	7.12 ± 1.36	
Distant metastasis			
M0	14	4.96 ± 1.44	0.346
M1	32	7.12 ± 1.36	
Responses to chemotherapy			
PR	6		
Befor-chemo		8.53 ± 1.93	0.189
After-chemo		5.63 ± 0.72	
SD	9		
Befor-chemo		5.93 ± 0.94	0.76
After-chemo		6.44 ± 1.35	
PD	9		
Befor-chemo		6.02 ± 1.68	0.053
After-chemo		23.05 ± 7.44	

**Table 4:** Clinicopathological parameters and responses to chemotherapy with urine TFF3 in colorectal cancer patients

**Item**	**Cases (n)**	**Urine TFF3 (ng/ml)**	***P*-value**
Age			
≤60	37	5.71 ± 0.94	0.088
>60	20	3.99 ± 0.89	
Gender			
Male	37	5.17 ± 0.93	0.558
Female	20	6.04 ± 1.03	
Site			
Colon	38	4.84 ± 0.64	0.201
Rectum	19	6.75 ± 1.66	
Degree of differentiation			
Well-moderately	47	5.67 ± 0.84	0.558
Poorly	10	4.58 ± 0.56	
Stage		(I-III *VS* IV)	0.222
I-II	9	3.72 ± 1.16	
III	11	4.60 ± 1.58	
IV	37	6.16 ± 0.92	
Lymphatic metastasis			
N(-)	9	2.92 ± 1.00	0.164
N(+)	14	5.57 ± 1.32	
Distant metastasis			
M0	20	4.20 ± 0.99	0.184
Responses to chemotherapy			
PR	8		
Befor-chemo		9.18 ± 3.06	0.076
After-chemo		3.06 ± 0.93	
SD	17		
Befor-chemo		5.95 ± 1.16	0.76
After-chemo		8.83 ± 3.35	
PD	4		
Befor-chemo		3.26 ± 0.87	0.13
After-chemo		9.87 ± 3.68	

## Discussion

Gastric and colorectal cancer are the two most common malignancies worldwide [19]. Despite developed strategies of chemotherapy and radiotherapy, the curative treatment for gastric and colorectal cancer is the surgical resection of primary tumors at early stages [20,21]. Certain patients with gastric and colorectal cancer, even with the same TNM stage, have different prognoses and treatment responses. Therefore, new biological markers for early detection and predictors of prognosis for gastric and colorectal cancer are urgently needed in clinical work.

Secreted proteins play an important role in cell signaling, communication and migration [22,23]. The secreted protein TFF3 has malignant characteristics to promote the invasion of tumor cells by acting both directly on malignant cells and indirectly on the vasculature [24]. TFF3 is upregulated in most human malignancies, and the protein’s expression is correlated with a highly aggressive phenotype and poor prognosis [15,25,26]. However, TFF3 has been reported by several investigators to have conflicting roles in these regards [8,13,27,28].

Several earlier clinical studies identified serum TFF3 as a new marker for gastric cancer [13,29]. In previous study, a serum TFF3 level greater than 7 ng/ml indicated higher sensitivity in predicting the presence of gastric cancer [30,31]. In this study, we found that serum and urine TFF3 levels of the patients with gastric and colorectal cancer were significantly higher that in healthy individuals. High serum levels of TFF3 were significantly correlated with distant metastasis and an advanced stage in both types of cancer. This suggest that high TFF3 expression was significantly correlated with vein invasion, and advanced stage. TFF3 may play an important role in promoting gastrointestinal cancer development, progression and dissemination. Our data have shown that decreased serum TFF3 levels were significantly correlated with responses to chemotherapy in both the gastric and the colorectal cancer PR groups. However, no significant changes were observed in patients with PD or SD after chemotherapy. Thus, our results suggested that serum TFF3 may be a potential useful marker for patients with gastric and colorectal cancer and their metastases. It may be a pharmacodynamics marker of responses to chemotherapy in both gastric and colorectal cancer PR patients.

To investigate whether urine TFF3 can also be used as a biomarker for gastric and colorectal cancer detection, we analyzed urine and serum TFF3 levels at the same time. Interestingly, urine TFF3 levels in the patients with gastric and colorectal cancer were significantly elevated compared with the levels of the healthy individuals. However, the combination of serum and urine data could not significantly improve the detection of both cancers compared with serum or urine TFF3 data alone, and urine TFF3 levels did not show a significant correlation with serum levels. Our data also did not show a significant difference for the urine TFF3 level based on clinical stage, lymphatic metastasis, the patient’s gender or age, the site or degree of tumor differentiation or responses to chemotherapy for gastric or colorectal cancer. However, urine TFF3 levels were significantly negatively correlated with the GFR in both the gastric and the colorectal cancer groups. This result suggests that the origin of urine TFF3 in gastric and colorectal cancer is much more complicated than the serum levels. TFF3 is a small peptide hormone produced by epithelial cells in multiple tissues, including the kidney. The decreased GFR in acute and chronic kidney diseases is caused by pathological damage to the kidney structure [32]. Urine TFF3 is a sensitive and specific biomarker for tubular injury used to detect early renal dysfunction [33,34]. Decreases in urinary TFF3 are associated with proximal tubular injury and correlate well with the severity of renal histopathological lesions [35,36]. Our results suggested that urine TFF3 is a different indicator to serum levels and that multiple factors can affect urine TFF3 levels, such as the secretion of cancer, tubular secretion and the GFR.

In summary, our data indicated that serum TFF3 can be applied as an effective biomarker for the detection of tumor stages and distant metastasis and as a predictor of responses to chemotherapy in both gastric and colorectal cancer. Urine TFF3 is a different indicator to serum levels and could also be a biomarker for the early detection of renal dysfunction.

## Conclusions

In summary, our data indicated that serum TFF3 can be applied as an effective biomarker for the detection of tumor stages and distant metastasis and as a pharamcodynamic marker of responses to chemotherapy in gastrointestinal cancer. Urine TFF3 is a different indicator to serum levels and could also be a biomarker for the early detection of renal dysfunction.

## Abbreviations

TFF: Trefoil factor family; ITF: Intestinal trefoil factor; SD: Stable disease; PD: Progressive disease; PR: Partial response; CR: Complete response; GFR: Glomerular filtration rate.

## Competing interests

The authors declare that they have no competing interests.

## Authors’ contributions

JLR and BG designed the experiments; LX, YPL and CXX performed the research; and LX and BG wrote the paper. All the authors revised the manuscript and approved the final version.

## Pre-publication history

The pre-publication history for this paper can be accessed here:

http://www.biomedcentral.com/1472-6890/14/26/prepub
